# Understanding resilience: Lifestyle-based behavioral predictors of mental health and well-being in community-dwelling older adults during the COVID-19 pandemic

**DOI:** 10.1186/s12877-024-05251-3

**Published:** 2024-08-12

**Authors:** Mikael Anne Greenwood-Hickman, Lily N. Shapiro, Shirley Chen, Paul K. Crane, Laura B. Harrington, KatieRose Johnson, Andrea Z. LaCroix, Liam G. Lane, Susan M. McCurry, Pamela A. Shaw, Dori E. Rosenberg

**Affiliations:** 1 Kaiser Permanente Washington Health Research Institute, 1730 Minor Ave, Ste. 1360, Seattle, WA 98101 USA; 2 Department of Medicine, University of Washington, Seattle, WA USA; 3 Department of Epidemiology, University of Washington, 1959 NE Pacific Street, Seattle, WA 98195 USA; 4 Department of Health Systems Science, Kaiser Permanente Bernard J. Tyson School of Medicine, 98 S. Los Robles Ave, Pasadena, CA 91101 USA; 5 Herbert Wertheim School of Public Health and Human Longevity Science, University of California San Diego, 9500 Gilman Dr, La Jolla, CA 92093 USA; 6 School of Nursing, University of Washington, 1959 NE Pacific Street, Seattle, WA 98195 USA

**Keywords:** Physical activity, Sleep, Depression, Social support, Fatigue, COVID-19 pandemic, Resilience, Lifestyle

## Abstract

**Background:**

Changes in sleep, physical activity and mental health were observed in older adults during early stages of the COVID-19 pandemic. Here we describe effects of the COVID-19 pandemic on older adult mental health, wellbeing, and lifestyle behaviors and explore predictors of better mid-pandemic mental health and wellbeing.

**Methods:**

Participants in the Adult Changes in Thought study completed measures of lifestyle behaviors (e.g., sleep, physical activity) and mental health and wellbeing both pre-pandemic during regular study visits and mid-pandemic via a one-time survey. We used paired t-tests to compare differences in these measures pre- vs. mid-pandemic. Using multivariate linear regression, we further explored demographic, health, and lifestyle predictors of pandemic depressive symptoms, social support, and fatigue. We additionally qualitatively coded free text data from the mid-pandemic survey for related comments.

**Results:**

Participants (N = 896) reported significant changes in mental health and lifestyle behaviors at pre-pandemic vs. mid-pandemic measurements (p < 0.0001). Qualitative findings supported these behavioral and wellbeing changes. Being male, never smoking, and lower pre-pandemic computer time and sleep disturbance were significantly associated with lower pandemic depressive symptoms. Being partnered, female, never smoking, and lower pre-pandemic sleep disturbance were associated with higher pandemic social support. Pre-pandemic employment, more walking, less computer time, and less sleep disturbance were associated with less pandemic fatigue. Participant comments supported these quantitative findings, highlighting gender differences in pandemic mental health, changes in computer usage and physical activity during the pandemic, the value of spousal social support, and links between sleep disturbance and mental health and wellbeing. Qualitative findings also revealed additional factors, such as stresses from personal and family health situations and the country’s concurrent political environment, that impacted mental health and wellbeing.

**Conclusions:**

Several demographic, health, and lifestyle behaviors appeared to buffer the effects of the COVID-19 pandemic and may be key sources of resilience. Interventions and public health measures targeting men and unpartnered individuals could promote social support resilience, and intervening on modifiable behaviors like sleep quality, physical activity and sedentary activities like computer time may promote resilience to fatigue and depressive symptoms during future community stressor events. Further research into these relationships is warranted.

**Supplementary Information:**

The online version contains supplementary material available at 10.1186/s12877-024-05251-3.

## Background

In March 2020, the United States began implementing state-level public health mitigation measures to limit the spread of COVID-19. Adults aged 65 years and older, particularly those with chronic health conditions, were at the highest risk for contracting and dying from COVID-19 infection [1, 2], making adherence to these mandates particularly important for this population. However, older adults were already at an increased risk for social isolation, loneliness, and high levels of sedentary behavior pre-pandemic [3–5], which were likely to be exacerbated by mandated social distancing measures.

More recently, there has been mounting evidence of the impacts of the COVID-19 pandemic on older adults’ daily lives, mental health, and activities, including lifestyle behaviors such as physical activity, sedentary behavior, and sleep. Specifically, studies from older adult populations across the globe have documented higher depressive symptoms, stress, and anxiety [6–8]; lower social support and engagement [9–12]; interruptions to daily life, living arrangements, and employment [13–15]; changes to physical activity and sedentary behavior patterns [16–18]; and lower sleep quality [19, 20]. Outside the context of the pandemic, lower physical activity, higher sedentary behavior, and worse sleep quality have been associated with worsened depressive symptoms and other measures of wellbeing [21–24]; changes to these lifestyle behaviors from the pandemic, particularly if sustained long-term, could be associated with deleterious mental health and wellbeing outcomes for older adults.

It is incompletely understood whether demographic and pre-pandemic lifestyle behavioral predictors might be protective of wellbeing during events like the COVID-19 pandemic. We are aware of only two studies in older adults that explored cross-sectional associations between self-reported pandemic lifestyle behavior patterns and depression and anxiety. Robbins et al. documented that self-reported low levels of sleep quality, changes in sleep time, greater amounts of TV time, and lower amounts of walking during the pandemic were associated with more feelings of depression and anxiety [7]. Similarly, Amerio and colleagues documented lower levels of sleep quality and higher levels of depressive symptoms and anxiety, further suggesting that these outcomes were worse for women and among those who had lower levels of physical activity during the pandemic [25]. These studies were cross-sectional, with no pre-pandemic measure, and only included data collected within the first months of the pandemic period. Pre-pandemic assessment of these behavioral and wellbeing factors is needed to assess change during the pandemic period and establish temporality in possible protective associations observed. Additionally, to our knowledge, no studies have assessed the sustained impacts and longer-term consequences for the mental health of older adults beyond a year into the pandemic period.

Here, we apply a mixed methods approach to leverage data from the Adult Changes in Thought (ACT) study collected at regular study visits pre-pandemic and during a one-time survey in 2021, just over 1 year after the March 2020 initiation of public health mitigation measures in the US. Our specific objectives were to 1) characterize within-person changes in self-reported lifestyle behaviors (physical activity, sedentary time, sleep) and mental health and wellbeing measures (depression, social support, fatigue) from pre-pandemic to mid-COVID-19 pandemic; 2) evaluate demographic, health, and lifestyle behavior predictors of depressive symptoms during the pandemic, fatigue, and social support outcomes; and 3) explore qualitative findings related to behavioral and mental health coping and resilience from free response text. We hypothesized that higher levels of healthful lifestyle behaviors (e.g., more physical activity, less sedentary behavior, better sleep quality) pre-pandemic would be associated with better mental health and wellbeing measures mid-pandemic.

## Methods

### Setting

**Fig. 1:**
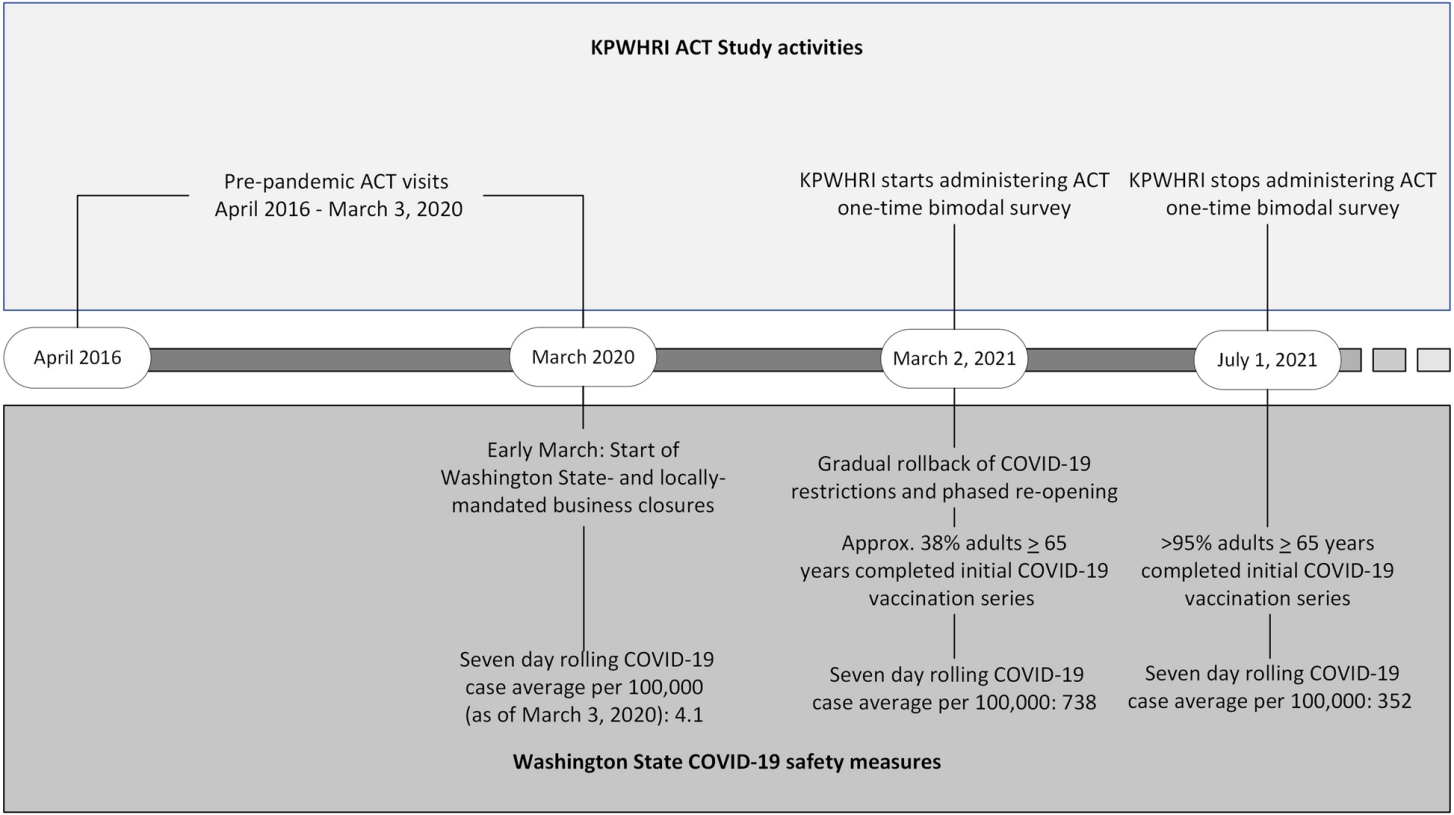
Timeline of key ACT measurement activities and pandemic milestones in the Seattle, WA area References: [26–30]

The ACT study is an ongoing epidemiologic cohort study conducted at the Kaiser Permanente Washington Health Research Institute in King County (Seattle), Washington, USA. All procedures were approved by the Kaiser Permanente Interregional institutional review board, and participants provided full written informed consent. Pre-pandemic data were obtained through in-person biennial measurements (performed face-to-face in a research clinic or the participant’s home based on individual preference) and a self-administered paper survey returned by mail after the visit between 2016 and 2020. Beginning in early March 2020, Washington State implemented state and locally mandated business closures and encouraged residents to stay home and practice social distancing. These restrictions remained in place until early 2021, at which point restrictions were gradually eased and a phased re-opening plan was initiated [26]. Mid-pandemic data was obtained via a one-time survey either self-administered online or administered by study staff over the phone during the COVID-19 pandemic between March 2, 2021 and July 1, 2021. By March 2, 2021, when the ACT pandemic survey was initiated, a widespread COVID-19 vaccination campaign was underway for older adults, and approximately 38% of adults over age 65 years had been vaccinated [27]. By July 1, 2021, when survey fielding completed, > 95% of older adults in King County Washington had completed an initial COVID-19 vaccination series [27]. See Fig. 1 for a timeline of key pandemic-related milestones in the greater Seattle, Washington area.

### ACT study overview

ACT is an ongoing, prospective cohort study initiated in 1994 [31, 32]. ACT invites a random sample of Kaiser Permanente Washington (KPWA) members aged ≥ 65 years without dementia to participate. KPWA is an integrated health care delivery system, from which members receive the majority of their care. Study participants undergo biennial follow-up visits to screen for incident dementia. Participants are followed until dementia onset, study disenrollment, or death. Starting in 2000 an expansion cohort was recruited to increase the study size, and in 2005 continuous enrollment of participants began to ensure a stable active cohort of approximately 2000 older adults. Study visits are completed at a central research clinic or at the participant’s home, based on participant need and preference [33]. Biennial visit procedures include cognitive testing, physical performance testing, and a variety of self-reported demographic and health measures collected via in-person interview by study staff [32]. Beginning in 2016 an additional self-reported survey was added to ACT visits to collect additional measures of well-being (mental health, quality of life, etc.), and lifestyle behaviors (physical activity, sedentary behavior, and sleep) [34]. All demographic and pre-pandemic measures presented in this analysis were collected as part of routine ACT study visits between April 27, 2016 and March 3, 2020.

### ACT COVID-19 pandemic survey procedures

During the COVID-19 pandemic (March 2, 2021 through July 1, 2021), participants were invited via postal mail to complete a one-time web survey or to arrange contact with a study staff member and complete the one-time survey by phone. Survey items included a series of closed-ended items on topics related to lifestyle behaviors (physical activity, sleep, and sedentary behavior), and mental and physical health during the pandemic. Wherever possible, items and scales used in the standard ACT Biennial Visit were used in this survey to facilitate comparison with prior responses. This survey also included free-text, open-ended items where participants could share further details on their experiences during the pandemic. Participants were mailed $10 cash with a thank you letter upon completion.

### Predictors

#### Demographic predictors

Demographic predictors were self-reported at the participants’ most recent study visit pre-pandemic and included age (years, continuous), sex (male vs. female), race (reported as White, Black, Asian, and other races including multiple race groups; consolidated as self-reported White vs. all other race groups for analysis due to limited sample size), ethnicity (Hispanic vs. non-Hispanic), years of education (continuous), retirement status (currently working for pay vs. not), marital status (currently married or partnered vs. single/divorced/widowed), and living arrangement (living alone vs. living with others).

#### Health and function predictors

Health and function predictors included: smoking status assessed by self-report at the pre-pandemic ACT study visit (never, current, former smoker; dichotomized as ever vs. never smoker for modeling due to limited sample size); body mass index (BMI) calculated from height and weight measured at the same pre-pandemic ACT study visit (kg/m^2^; continuous); Charlson Comorbidity Index (CCI continuous, range 0–24, high score suggests higher morbidity burden) scores from electronic health records in the year prior to the pre-pandemic ACT visits [35, 36]. Self-reported physical function measured via two summary scores. The first was an Activities of Daily Living (ADL) summary that reflected the number of six distinct ADLs the participant endorsed having difficulty with: walking around the house, getting out of bed or a chair, feeding oneself, dressing oneself, bathing/showering oneself, and getting to or using the toilet (continuous; range 0–6, higher scores denote difficulty with more tasks). A second summary measure represented difficulty with five Instrumental ADLs (I-ADLs; continuous; range 0–5, higher scores denote difficulty with more tasks): light housework, shopping for personal items, preparing meals, managing finances, and using the telephone [37–41].

#### Lifestyle behavior predictors

Pre-pandemic lifestyle behavior predictors were self-reported via written survey immediately following the most recent study visit pre-pandemic. We selected reliable and valid measures that were specifically selected for their appropriateness in older populations. Physical activity was reported as days per week engaging in brisk walking, an item taken from the validated Community Healthy Activities Model Program for Seniors (CHAMPS) questionnaire [42, 43]. Daily TV time and computer time were collected using items from the validated Sedentary Behavior Questionnaire [44, 45]. Participants estimated typical daily time in each activity according to the following categories: “None”, “Less than 30 min”, “30–60 min”, “1–2 h”, “2–3 h”, “3–4 h”, “4–5 h”, “5–6 h”, “6–7 h”, “7–8 h”, and “8 + hours”. Total daily sedentary behavior was assessed via the following separate question adapted from the Women’s Health Initiative Objective Physical Activity and Cardiovascular Health Study [46]: “During a usual day and night, about how many hours do you spend sitting?” Categorical response options included: “Less than 4 h”, “4–5 h”, “6–7 h”, “8–9 h”, “10–11 h”, “12–13 h”, “14–15 h”, and “16 or more hours”. For analysis, the midpoint value of each selected response range was taken (e.g., for “1–2 h”, a value of 1.5 h) to estimate a daily average time in hours for each activity. For responses indicating the maximal category (i.e., 8 + or 16 + hours), the assigned value was truncated at 8 or 16 h, respectively.

Sleep quality was measured using the reliable and valid 8-item Patient-Reported Outcomes Measurement Information System (PROMIS) Sleep Disturbance scale [47, 48]. For analysis, raw scores were converted to T-scores and standard error estimates according to PROMIS scoring tables (continuous, higher scores indicate more sleep disturbance and lower quality sleep). Pre-pandemic levels of each outcome measure (depressive symptoms, social support, and fatigue) were measured using the same scales described under “Outcomes”.

### Quantitative outcome measures

Outcomes of interest include levels of three key measures of mental health and wellbeing collected on the ACT pandemic survey in 2020. Measures were also collected as part of regular ACT biennial visits pre-pandemic and were specifically selected for their appropriateness in an older adult population. Depressive symptoms were measured using the reliable and validated Center for Epidemiologic Studies Depression 10-item Scale (CES-D-10; scores range 0–30 and scores ≥ 10 indicate significant depressive symptoms vs. scores < 10) [49, 50]. Social Support was measured using a shortened, 6-item version of the validated Interpersonal Support Evaluation List [51] (ISEL; scores range 6–24, higher scores indicate more social support). Energy and fatigue were measured using the 4-item vitality scale from the reliable and valid Rand 36-item Short Form (Rand SF-36; scores range 0–100, higher scores indicate more energy and less fatigue) [52, 53].

### Qualitative data

All free-text narrative data included were derived from participant typed (web survey) or dictated (phone survey) responses to the following open-ended questions: “Have you found any new ways to be physically active during the COVID-19 pandemic? If yes, what are they?”; “What other technologies have you used to stay in touch with others during the stay-at-home order?”; and “Is there anything else you feel we should know about how your lifestyle has changed because of the COVID-19 pandemic?”.

### Statistical analyses

All data underwent basic review and cleaning for invalid and missing values as part of standard ACT study procedures. We computed descriptive statistics (frequency, mean, standard deviation [SD]) for pre-pandemic baseline demographic, health/function, and lifestyle behavior measures. We further calculated descriptive statistics (mean, SD) for both pre-pandemic and pandemic measures of each outcome and behavioral predictor of interest, and reviewed histograms of each outcome variable to assess approximate normality. We calculated pre-pandemic to pandemic change scores for each behavioral and outcome measure by subtracting pre-pandemic scores from pandemic scores. We used two-tailed paired T-tests to test for statistically significant differences between pre-pandemic and pandemic levels of each measure.

In order to explore potential predictors of pandemic mental health and wellbeing, we sequentially fit two multivariate linear regression models for each outcome to separately evaluate demographic and behavioral predictors of pandemic depressive symptoms, social support, and fatigue using a complete case approach. First, in order to explore the relationship between demographic and health predictors alone, we fit Model 1, which included demographic characteristics (age, sex, race, ethnicity, education, retirement status, marital status, and living arrangement) and pre-pandemic health and function measures (BMI, CCI, smoking status, difficulty with ADLs, and difficulty with IADLs) only. Then, to understand if pre-pandemic behavioral measures further explained variation in the outcome not captured by the demographic and health covariates, we separately fit Model 2, which included all variables from Model 1 and further included self-reported pre-pandemic lifestyle behavioral measures identified a priori (days briskly walking, daily total sitting time, daily TV time, daily computer time, and sleep quality). To account for possible seasonal variation in some measures, the month of pandemic survey responses was also included in models as a covariate. To aid in interpretation of regression output, all continuous variables for which 0 was not a valid or probable value were centered on the sample mean.

To assess the correlation between pre-pandemic and pandemic levels of outcome measures, we calculated Pearson’s correlation coefficients between pre- and pandemic-measures. To assess potential concerns for collinearity and other violations of regression model assumptions, we additionally calculated Pearson’s correlation coefficients between all independent variables, assessed variance inflation factors for all covariates, and repeated models using robust standard error calculations. To assess the impact of baseline adjustment, we repeated Model 2, additionally adjusting for baseline values of the applicable outcome measure of interest and a measure of the time (in years) between pre-pandemic and pandemic measures. All statistical analyses were completed in Stata, version 15.1 (StataCorp LLC, College Station, TX).

### Qualitative analysis

We primarily used a triangulated mixed methods [54, 55] approach for the qualitative analysis, in which data from the ACT pandemic survey free text questions were used to add context and depth to the quantitative results. As with other mixed methods studies [56–58], we first used quantitative methods to identify factors associated with our outcomes of interest. We then turned to the free text data to perform a qualitative analysis in order to better understand these factors. We developed a list of a priori codes of interest, based largely on the quantitative results, and drawn in part from an existing codebook from a recent study [13]. Because we were interested in elaborating on our quantitative findings specifically, we primarily utilized a deductive coding approach but allowed for inductively generated codes to arise during the coding process as appropriate, allowing for an investigation of qualitative data pertaining to pandemic-related impacts to daily life, health, and coping strategies. Text data management and coding was conducting using Atlas.ti version 9 (Atlas.ti Scientific Software Development GmbH, Berlin, Germany). We initially used the Text Search feature in Atlas.ti to perform key word searches of the free text data and assigned codes for broad topical categories of interest: technology use, TV watching, sleep, sitting, physical activity & exercise, depressive symptoms, loneliness, social support, fatigue, and anxiety and stress. Search terms were generated collaboratively by the two-person coding team trained in qualitative analysis (MAGH and LNS), based on synonyms and related words or word stems for each topic of interest, and were refined after an initial review of the free text data (see final search terms in SupplementalTable 1 of Additional File 1). All quotations tagged by the software were then reviewed by a single coder from the two-person team, and coders met to reconcile code assignments and iteratively refine the codebook. Once consensus was reached, and all data coded, we summarized the contextual insights gleaned from the free text data as they related to the significant predictors from the quantitative models in a mixed methods insights table for interpretation and discussion.

## Results

### Participant characteristics

Of the N = 1885 eligible participants at 2016 data collection, the ACT mid-pandemic survey was fielded to *n* = 1660 participants who met eligibility criteria at the time with a response rate of 77% (*n* = 1276 responses). A total of N = 896 ACT participants completed and returned a biennial survey at least once between 2016 and March 2020 (pre-pandemic) and completed the ACT mid-pandemic survey in 2021, representing a combined response rate across both surveys of 47.5%. Characteristics of ACT participants who responded to both surveys and of ACT participants who were eligible but did not respond to one or both survey (non-responders) are described in Table 1. The mean age in this sample was 77.2 years (SD = 6.6) at their pre-pandemic measurement, with an average (SD) of 3.0 (1.3) years between pre-pandemic and pandemic survey responses. 57.6% of participants were female, 89.3% self-reported White race, 98.3% self-reported non-Hispanic ethnicity, and the sample was highly educated (mean 17.1 [standard deviation 2.7] years education). Most participants were married or partnered (59.2%); lived with others (65.0%); and did not currently work for pay at the time of their pre-pandemic study visit (82.8%). The population was also generally healthy, with low smoking rates (1.7% current, 5.6% former smokers) and few impairments in activities of daily living. Non-responders were older on average, a smaller proportion self-reported White race, had fewer years of schooling on average, had higher proportions who were single and who lived alone, and had more chronic health conditions and impairments to ADLs on average than responders.

**Table 1:** Baseline demographic and pre-pandemic characteristics of ACT participants responding to ACT COVID Survey (*N* = 896)

	**Responders**	**Non-Responders**
	*N* = 896	*N* = 989
	**mean (SD)**	**mean (SD)**
	**N (%)**	**N (%)**
**Age (years, pre-COVID)**	77.2 (6.6)	82.8 (7.9)
**Time between measures (years)**	3.0 (1.3)	N/A
**Sex**		
Male	380 (42.4%)	411 (41.2%)
Female	516 (57.6%)	578 (58.4%)
**Race**		
Asian	32 (3.6%)	39 (4.0%)
Black	15 (1.7%)	27 (2.7%)
Other race (including multiple races)	48 (5.4%)	45 (4.6%)
White	800 (89.3%)	873 (88.7%)
**Ethnicity**		
Non-Hispanic/Latino	879 (98.3%)	967 (98.3%)
Hispanic/Latino	15 (1.7%)	17 (1.7%)
**Education (years)**	17.1 (2.7)	16.0 (3.0)
**Currently work for pay**	154 (17.2%)	89 (9.3%)
**Marital Status**		
Single/Divorced/Widowed	360 (40.8%)	544 (57.1%)
Married/Partnered	523 (59.2%)	408 (42.9%)
**Living Arrangement**		
Live Alone	309 (34.5%)	415 (43.3%)
Live w/Partner, Relative, Friend	582 (65.0%)	497 (51.8%)
Live in Adult Family Home or Nursing Home	5 (0.6%)	47 (4.9%)
**Body Mass Index (BMI)**	27.1 (5.1)	26.6 (5.5)
**Charlson Comorbidity Index (CCI)**^a^	0.97 (1.50)	1.97 (2.33)
**Smoking Status**		
Never	831 (92.8%)	916 (96.0%)
Former	50 (5.6%)	17 (1.8%)
Current	15 (1.7%)	21 (2.2%)
**Number ADLs with Difficulty**		
None	724 (80.8%)	587 (62.3%)
1 or more	172 (19.2%)	355 (37.7%)
**Number IADLs with difficulty**		
None	768 (86.2%)	614 (66.7%)
1 or more	123 (13.8%)	306 (33.3%)

Note: Some categories may not sum the listed N due to missingness in a covariate. For Responders (Race *n* = 1; Hispanic ethnicity *n* = 2; marital status *n* = 13; BMI *n* = 24; CCI *n* = 40; I-ADLs *n* = 5). For non-responders (Race *n* = 5; Hispanic ethnicity *n* = 5; work for pay *n* = 29; marital status *n* = 37; living arrangement *n* = 30; BMI *n* = 118; CCI *n* = 82; smoking status n35; ADLs *n* = 47; I-ADLs *n* = 69)

*Abbreviations*: *SD *standard deviation, *BMI *body mass index, *CCI *Charlson Comorbidity Index, *ADLs *activities of daily living, *IADLs *instrumental activities of daily living

^a^CCI is set to missing for individuals without current enrollment data or without a visit in the prior year

### Pre-pandemic vs. mid-pandemic descriptive findings

Table 2 summarizes unadjusted pre-pandemic and mid-pandemic sample means for key mental health and well-being outcomes and behavioral predictors of interest. Mean age at pandemic survey completion was 80.2 years (6.6). Self-reported total daily sitting was just under 8 h on average pre-pandemic but was approximately half an hour shorter on average mid-pandemic. By contrast, individual measures of two key sedentary activities, daily TV time and daily computer time, were higher mid-pandemic by approximately half an hour each (2.6 h [1.7] vs. 3.0 h [2.0], and 1.9 h [1.6] vs. 2.3 h [1.8], respectively). Self-reported days per week walking briskly was more than half a day lower on average mid-pandemic (1.6 days [1.9] vs. 0.7 days [1.3]). Self-reported sleep quality was lower on average mid-pandemic, as indicated by a higher sleep disturbance score (46.7 [8.0] vs. 53.4 [7.9]). Depressive symptoms were higher on average mid-pandemic (3.7 [4.1] vs. 5.2 [4.9]), and more participants had a score of 10 or higher, which is clinically indicative of depression (9.7% vs. 20.8%) [49]. Both social support and energy and fatigue scores were higher on average mid-pandemic, indicating lower social support and more fatigue (22.7 [2.0] vs. 21.4 [2.9] and 62.3 [17.5] vs. 57.4 [19.9], respectively). All pre-pandemic vs. mid-pandemic comparisons were statistically significantly different (p < 0.0001).

**Table 2:** Comparison of unadjusted pre-pandemic and pandemic sample means of behavioral predictors and outcomes of interest^1^

	**Pre-Pandemic**	**Mid-Pandemic**	**Post–Pre Change**	**p-value**^**2**^
	mean (SD)	mean (SD)	mean (SD)	
**Age (years)**	77.2 (6.6)	80.2 (6.6)	N/A	N/A
**Daily Total Sitting Time (h)**^**3**^	7.9 (2.9)	7.4 (3.0)	-0.5 (3.1)	< 0.0001
**Daily TV time (h)**^**3**^	2.6 (1.7)	3.0 (2.0)	0.4 (1.8)	< 0.0001
**Daily Computer Time (h)**^**3**^	1.9 (1.6)	2.3 (1.8)	0.4 (1.7)	< 0.0001
**Days/week Walking for Exercise**	1.6 (1.9)	0.7 (1.3)	-0.6 (1.8)	< 0.0001
**Sleep Disturbance (PROMIS T-score)**	46.7 (8.0)	53.4 (7.9)	6.7 (6.5)	< 0.0001
**Social Support Scale (ISEL) Score**	22.7 (2.0)	21.4 (2.9)	-1.3 (2.2)	< 0.0001
**Depressive Symptoms (CES-D 10-item Score)**	3.7 (4.1)	5.2 (4.9)	1.5 (4.1)	< 0.0001
**Energy/Fatigue (SF-36 sub-scale score)**	62.3 (17.5)	57.4 (19.9)	-5.0 (15.3)	< 0.0001

*Abbreviations: SD *standard deviation, *PROMIS *Patient-Reported Outcomes Measurement Information System, *ISEL *Interpersonal Support Evaluation List, *CES-D *Center for Epidemiologic Studies Depression, and SF-36 Rand 36-item Short Form

^1^The included N in each summary varies from the total of *N* = 896 due to missingness in each variable from skipped or incomplete survey items. The number missing for each measure is as follows: total daily sitting *n* = 48; daily tv time *n* = 20; daily computer time *n* = 23; days briskly walking *n* = 48; sleep disturbance *n* = 22; ISEL *n* = 46; CES-D *n* = 24; SF-36 *n* = 9

^2^p-values derived from a paired t-test (continuous) of pre- vs. mid-pandemic self-reported measures of each item. *P* < 0.05 was considered statistically significant

^3^Self-reported time was reported in categorical variables by approximate number of total hours (h) (e.g. 1–2 h, 2–3 h, etc.) and transformed to an approximate total number of hours by assigning the midpoint of the chosen range. More details available in the Methods section.

### Predictors of pandemic mental health & wellbeing

The sample size included in each model varied due to missingness of included variables, but complete case samples for each model were similar to the full sample in terms of demographic characteristics, health status, and sample means for key behavioral predictors and outcomes (SupplementalTable 2 of Additional File 1). After considering model fit statistics for Models 1 and 2 for all outcomes of interest, we determined that Model 2, which additionally included lifestyle behavioral predictors, was the more complete model (Model 1 vs. Model 2 adjusted R-squared values: Depressive Symptoms 0.061 vs. 0.148; Social Support 0.035 vs. 0.035; fatigue 0.110 vs. 0.165). Therefore, results for all outcomes of interest from Model 1 (demographic and health predictors only) can be found in SupplementalTable 3 (see Additional File 1), and we present fully adjusted results including pre-pandemic lifestyle behavior predictors from Model 2 are displayed in Table 3.

**Table 3:** Demographic, health, and lifestyle predictors of pandemic depressive symptoms, social support and fatigue (Model 2)

	**A. Pandemic Depressive Symptoms (CES-D)** *N* = 691			**B. Pandemic Social Support (ISEL)** *N* = 675			**C. Pandemic Fatigue**^**4**^** (SF-36)** *N* = 703		
	β	95% CI		β	95% CI		β	95% CI	
**DEMOGRAPHIC FACTORS**									
**Pre-pandemic Age** (y)^**1**^	-0.03	-0.09	0.03	0.04	0.00	0.08	-0.13	-0.37	0.12
**Male Sex** (vs. female)	**-0.71**	**-1.40**	**-0.02**	**-0.51**	**-0.97**	**-0.06**	2.44	-0.44	5.33
**Black, Asian, Other race** (vs. White)^2^	-0.56	-1.64	0.52	-0.25	-0.96	0.47	2.96	-1.54	7.46
**Hispanic Ethnicity** (vs. not)	1.32	-1.29	3.93	0.27	-1.54	2.08	-7.47	-18.51	3.58
**Years Education**^**1**^	0.11	-0.03	0.25	0.00	-0.09	0.09	-0.25	-0.83	0.34
**Currently Work for Pay** (vs. not)	-0.86	-1.78	0.05	-0.10	-0.70	0.51	**4.00**	**0.17**	**7.84**
**Married/Partnered** (vs. not)	-0.67	-1.92	0.57	**0.84**	**0.00**	**1.68**	-1.46	-6.69	3.77
**Live Alone** (vs. live with others)	-0.22	-1.48	1.03	0.28	-0.56	1.13	-0.99	-6.25	4.27
**HEALTH FACTORS**									
**Body Mass Index** (kg/m^2^)	0.01	-0.06	0.07	0.01	-0.04	0.05	**-0.32**	**-0.60**	**-0.03**
**Charlson Comorbidity Index**	0.20	-0.03	0.43	-0.03	-0.19	0.12	**-1.15**	**-2.13**	**-0.18**
**Ever Smoker** (vs. never)	**2.20**	**0.93**	**3.47**	**-1.08**	**-1.92**	**-0.24**	**-6.89**	**-12.21**	**-1.57**
**Number ADLs with difficulty**	0.34	-0.31	0.98	-0.13	-0.56	0.30	**-3.08**	**-5.74**	**-0.42**
**Number IADLs with difficulty**	0.27	-0.40	0.93	0.14	-0.31	0.58	**-3.71**	**-6.49**	**-0.92**
**LIFESTYLE BEHAVIORS**									
**Pre-Pandemic Total Daily sitting** (h)^**1,3**^	-0.05	-0.17	0.07	-0.04	-0.12	0.04	0.04	-0.47	0.55
**Pre-Pandemic TV time** (h)^**1,3**^	0.06	-0.15	0.27	-0.11	-0.24	0.03	-0.80	-1.68	0.07
**Pre-Pandemic Computer Time** (h)^**1,2**^	**0.27**	**0.04**	**0.49**	-0.07	-0.22	0.08	**-1.13**	**-2.08**	**-0.18**
**Pre-Pandemic Walking** (days)^**1**^	-0.07	-0.25	0.11	0.02	-0.10	0.14	**1.09**	**0.33**	**1.85**
**Pre-Pandemic Sleep Disturbance**^**1**^ (T-score)	**0.19**	**0.15**	**0.23**	**-0.04**	**-0.07**	**-0.01**	**-0.53**	**-0.71**	**-0.35**
**Survey month**	0.07	-0.30	0.43	0.14	-0.11	0.38	-0.01	-1.56	1.53
**Constant**	**5.41**	**3.32**	**7.51**	**20.61**	**19.21**	**22.01**	**58.84**	**50.08**	**67.61**

*Abbreviations*: *CES-D *Center for Epidemiologic Studies Depression [49, 50], *ISEL *Interpersonal Support Evaluation List [51], *SF-36 *Rand 36-item Short Form [52, 53], *BMI *body mass index, *CCI *Charlson Comorbidity Index [35, 36], *ADLs* activities of daily living, *IADLs* instrumental activities of daily living [37–41], h hours, y years

^1^Variables were centered at the sample mean value for modeling to allow interpretation of the intercept values at those sample means. Centering values are as follows: age=77.17746 years, years education=17.08817, BMI=27.06915, CCI= 0.9731308 , pre-pandemic total sitting=7.893868, pre-pandemic TV time=2.592466, pre-pandemic computer time=1.851947,  pre-pandemic walking=1.558962, and pre-pandemic sleep disturbance=46.78341

^2^Participants identifying as Black, Asian, or other race groups were grouped and compared with White participants for modeling purposes due to small sample size in individual race categories

^3^Self-reported time was reported in categorical variables by approximate number of total hours (h) (e.g., 1-2 h, 2-3 h, etc.) and transformed to an approximate total number of hours as described in the methods section

^4^Higher scores on the SF-36 denote more favorable health states. Here, higher scores indicate less fatigue (more energy).

#### Depressive symptoms

In model 1 (*N* = 792; SupplementalTable 3A of Additional File 1), without adjustment for lifestyle behaviors, male gender and being married or partnered were associated with significantly lower pandemic CES-D scores, while ever smoking status and difficulty with ADLs were associated with significantly higher pandemic CES-D scores. In model 2 (*N* = 691; Table 3A), which further adjusted for behavioral measures of interest, male gender was associated with lower pandemic CES-D score (β = -0.71; -1.40, -0.02) and smoking status was associated with a higher pandemic CES-D score (β = 2.20; 0.93, 3.47). Higher pre-pandemic computer time (β = 0.27; 0.04, 0.49), and higher pre-pandemic sleep disturbance (β = 0.19; 0.15, 0.23) were significantly associated with higher pandemic CES-D score. Marital status and difficulty with ADLs were not significant in Model 2. No associations were seen in either model for other predictors.

#### Social support

In model 1 (*N* = 774; SupplementalTable 3B of Additional File 1), older age was associated with more mid-pandemic social support, whereas male gender, current or former smoking status, and more difficulty with ADLs were associated with lower mid-pandemic social support. In model 2 (*N* = 675; Table 3B), being married or partnered (β = 0.84; 0.00, 1.68) was associated with higher pandemic social support, and male gender (β = -0.51; -0.97, -0.06), being a current or former smoker (β = -1.08; -1.92,-0.24), and higher pre-pandemic sleep disturbance (β = -0.04; -0.07, -0.01) were associated with lower mid-pandemic social support. However, age and difficulty with ADLs were not significant with adjustment for behavioral indicators. No associations were seen in either model for other predictors.

#### Fatigue

In model 1 (*N* = 806; SupplementalTable 3C of Additional File 1), higher BMI, higher Charlson Comorbidity Index, current or former smoking status, and difficulty with ADLs and IADLs were all associated with lower pandemic SF-36 energy and fatigue scores (i.e., lower energy and more fatigue). In Model 2 (*N* = 703; Table 3C), all five health and function measures were significant in Model 2 with similar effect sizes as seen in Model 1. Additionally, working pre-pandemic (β = 4.00; 0.17, 7.84) and more days walking pre-pandemic (β = 1.09; 0.33, 1.85) were associated with higher mid-pandemic SF-36 scores (less fatigue). Higher pre-pandemic computer time (β = -1.13; -2.08, -0.18) and more pre-pandemic sleep disturbance (β = -0.53; -0.71, -0.35) were associated with lower SF-36 scores (more fatigue). No associations were seen in either model for other predictors.

Pre- and mid-pandemic levels of depressive symptoms, social support, and fatigue were only moderately correlated (*r* = 0.60, *r* = 0.65, and *r* = 0.67, respectively). A correlation matrix of all independent variables used in modeling can be found in Supplemental Table 5. Correlations were low for all variable pairs, with the exception of marital status and living arrangement (*r* = 0.81, VIF = 3.4 and 3.5, respectively). VIF for all variables were all < 5, the common suggested cut-off for concern in all models [59, 60]. In sensitivity analyses using robust standard errors we note that the width of the 95% CI were very similar for each of the three outcome models (results not shown). Sensitivity analyses adjusting for pre-pandemic levels of each outcome of interest largely attenuated observed associations (results available in Supplemental Table 4).

### Qualitative findings

Qualitative results, derived from quantitative findings described above, are summarized in Table 4 along with supporting quotations.

**Table 4:** Qualitative descriptions of resilience and coping strategies during the COVID-19 pandemic by quantitative findings

Quantitative Finding		Qualitative Finding	
		**Description**	**Supporting Quotations**
**Depressive Symptoms**	**Gender**	Of those with the depression code, most (17/24) were female. People reported isolation as a driver of depressive symptoms, but equally emphasized politics and non-COVID-19 losses and health issues as drivers. This pattern was true for men and women, though women often used more intense language	***3907*** (f)—"Used to go to exercise 2x/week at community center which is now closed. Used to get respite from caregiving, but activity suspended. Used to visit friends, occasionally eat out. Life is far worse now." ***3330*** (m)—"Earlier in the year I had spates of mild depression, due partly to Covid stress and political situation…Hard to separate Covid effect from Trump effect!"
	**Computer Time**	Many people report feeling glad or "lucky" to find new ways (online, on the phone) to connect with friends and family, yet some also mention that this is a poor substitute for in-person contact	***2676***—"I could not go to the gym. zoom is much different than seeing my adult children and my grandchild" ***3326***—"I no longer work with kids,go to in person gatherings, volunteer places I used to or see most people in person including family and friends…I spend a lot of time on zoom and the phone and meditating…I learned a lot about myself but don't feel great about how to figure out what comes next."
	**Sleep**	Respondents often link poor sleep (both too much and too little) and depressive symptoms, and vice versa (better sleep with improved depressive symptoms)	***2750***—"Increase in sleeping. Depression. Lack of physical activity. Blob. Need socialization…" ***3481***—"Just before Covid lockdown, I had intense hip-back pain, decreasing the exercising I was doing. Then in April 2020, my husband got sick and died in May. I did no exercising for several weeks after that. My chronic tiredness and body pain (arthritis?) got worse. I have not gotten back to a decent exercise pattern since then: fatigue, pain, depression."
**Social Support**	**Gender**	Both men and women cited the inability to see friends and family, to go to church, and to attend community events and exercise classes as major losses, and as reasons for their loneliness. Some comments suggest, however, that men may have had thinner social networks prior to the pandemic	***3709*** (f)—"People in my age group are not able to see our grandchildren. My friends that are male are more isolated because they are not as comfortable communicating. They are not getting the help they need, being social." ***2696*** (f)—"Everything I was doing, the Y & working at the master gardeners and church, it all ended abruptly and all my social network was stopped. It was really devastating" ***3884*** (m) "Anxious to return to physical exercise at the YMCA. Now recognize a basic need and desire for companionship."
	**Marital Status**	People who had positive social support frequently cited their partners as sources of this support. Partners seem to fill gaps left in social activities by no longer being able to see friends or attend events in person. However, several people mentioned a partner’s worsening health status, or death, as a cause of stress and sorrow	***3005***—"…My salvation was having my husband for company and having my son and his family next door." ***3607***—"Wife and I have gotten to be better friends." ***3133***—"I feel very lucky that I have a wonderful relationship with my wife and have very supportive neighbors and family."
	**Sleep**	The direction of the relationship between sleep and social support is not completely clear from these data. However, people do link sleep quality to loneliness or stress	***2934***—"My previous "night owl" circadian rhythm (to sleep about 2 am) has morphed into a daily "night shift" schedule… This interferes with my spousal/family/social relations. I *could* sleep a more normal schedule. But I don't, because I enjoy the quiet, to-myself time." ***2791***—"I think I am better rested than before, more accepting of limitations on movement and contact, more thankful for the very good life I have. I very much miss regular physical contact with family members. Have had to make more effort to stay mentally and physically challenged."
**Fatigue**	**Retirement Status**	Respondents did not frequently discuss retirement or working status directly. However, a less structured daily routine for retired participants may have promoted disrupted sleep and activities that can disturb sleep (e.g., TV) for some Loss of social activities and engagement promoted feelings of low energy, which may have been accentuated among retired individuals or those who recently stopped working	***786***—"Find myself falling asleep because we stay up so late watching TV. Never took cat naps before….Grateful that… we didn't have young kids or having to work." ***994***—"Everyone lost energy because of lack of interactions."
	**Health Status**	Pain and other health conditions (e.g., sleep apnea) often interrupt sleep leading to daytime fatigue and tiredness. Treatment for severe illnesses (e.g., cancer) also cause fatigue and other side effects for some	***205***—"In the past 6 months I have experienced moderate to severe back and leg pain issues due to bulging disc, this has affected my daily activities and sleep" ***22***—"At the beginning of the pandemic I was recovering from lung cancer surgery. Energy and activity level were due to health, not the pandemic."
	**Walking**	Less physical activity and exercise may have promoted sleep difficulty and increased physical pain (e.g., from arthritis) and subsequent daytime fatigue Walking (outdoors and indoors) was a highly popular form of exercise during the pandemic. Many reported pre-pandemic walking routines became more regular. Others report walking greatly lessened due to interruption in pre-pandemic routines	***550***—"Before March of 2020 I would go to 2–3 plays a week and took classes; I saw friends, ate out, walked more. Since then, Zoom has been a stop gap means of "seeing" friends but not an adequate substitute nor comfort nor a means of being active. My arthritis is worse… When life returns to "normal" I do not know if I will have the mobility to enjoy the cultural life I did before…" ***565***—"walking more inside building" ***56***—"I continue to try and walk 10,000 steps each day." ***100***—"Much more walking with wife"
	**Computer Time**	Using the computer for work kept brain active and helped maintain social connection during the pandemic	***8***—"I still work part-time (CPA) and I moved home 3–6-2020. So much of my computer time has been work-related. That has been very helpful in keeping my brain active and staying connected to many people (clients and colleagues) during Covid. "
	**Sleep**	Interrupted and poor-quality sleep due to age, health, home environment causes daytime fatigue and tiredness	***1246***—"I am not sleeping 6 h a night but have concerns about sleep medications. I try to exercise and be productive but I feel tired and struggle with focus on projects."
**Other**	**N/A**	News consumption and worry about concurrent political climate and national current events interrupts sleep Living arrangement changes (e.g., moving) contribute to mental and physical strain, including fatigue Grief and worry from everyday, non-pandemic causes can interrupt sleep and cause fatigue (e.g., loss of spouse or loved one, worry about health decisions)	***655***—"I did experience a good deal of anxiety and worry during the pandemic, but it was mostly due to the [political situation]. For most of 2020 I experienced a daily headache as well as interrupted sleep from worry about … the country." ***517***—"Recent move caused more stress than I am used to. Also causing fatigue and backaches." ***723***—"My daily life has changed because my husband died … This has caused more of a change in my daily life than COVID-19 has. This has caused my depression, restless sleep, etc."

Note: As a significant predictor, findings related to smoking status were explored related to both depressive symptoms and social support. However, no comments specific to smoking were made by any participants in the sample.

#### Depressive symptoms

Qualitative findings were similar between men and women. In many cases, participants reported depression and other mental health issues attributed to circumstances other than the pandemic (e.g., loss/grief, non-COVID health conditions, political/social events). Participants often linked poorer sleep quality with depressive symptoms. Comments related to depressive symptoms and computer usage focused primarily on computer usage for exercise and social connection and did not directly address computer usage pre-pandemic.

#### Social support

Reports of lost in-person connection with friends, family, and community were common among both men and women respondents. Some comments suggested, however, that men may have had lower social support prior to the pandemic. Support from a partner/spouse was frequently cited in participant responses as a major source of connection, supporting quantitative findings.

#### Fatigue

Poor quality sleep or interrupted sleep from ongoing health issues (e.g., chronic conditions or severe illness treatment), stress, and other causes were clearly supported by the free text responses as drivers of daytime fatigue. Many participants discussed changes in physical activity and walking patterns, often attributed to sleep difficulty and daytime pain and fatigue. Some participants reported decreases in physical activity (e.g., no longer going to the gym or attending exercise classes), while others reported increases in overall activity or replacement activities (e.g., walking, gardening, cycling).

Notably, while not directly related to our measures of interest, many participants also mentioned feelings of stress and anxiety related to news consumption and worry about the US political climate and national current events ongoing concurrently with the pandemic. The comments provided little contextual data regarding the relationships between fatigue and computer time or retirement status, sleep and social support, or smoking behaviors.

## Discussion

Here, we explored changes in self-reported lifestyle behaviors and mental health and wellbeing outcomes for a sample of older adults between pre-pandemic and a mid-pandemic follow-up. Increases in sedentary behaviors, sleep disturbance, fatigue, and depressive symptoms and decreases in physical activity and social support were noted in this sample over a year into the pandemic period. Additionally, we used a mixed methods approach to explore predictors of better mental health and wellbeing at mid-pandemic, elucidating a number both modifiable and non-modifiable characteristics that may contribute to older adult resilience during stressor events like the COVID-19 pandemic.

In fully adjusted models, being male, never smoking, and having lower pre-pandemic computer time and sleep disturbance were significantly associated with lower depressive symptoms approximately 1 year into the pandemic period. This suggests that these traits and behaviors may have promoted resilience to depressive symptoms during the COVID-19 pandemic. Findings for gender, smoking, and sleep align with known correlates of depression in older adults outside the pandemic setting [61–63]. The findings for associations between sleep and depression were corroborated by our qualitative data. Associations of better sleep quality [7, 25] and male gender [25, 64] with lower depressive symptoms are consistent with cross-sectional studies from more acute phases of pandemic restrictions. Physical activity and TV time predictors observed as significant in Robbins et al., were notably not significant here. Future studies with more sensitive measurements of these behaviors are warranted.

Our finding that lower pre-pandemic computer time was protective against depressive symptoms runs counter to some non-pandemic literature among older adults that suggests that greater use of technology promotes social engagement and is associated with lower depressive symptoms [65]. Counter to the modeling results, qualitative findings suggested increased and new types of computer usage in this sample during the pandemic and emphasized participant perception of this digital connection as a lifeline of connection to social ties, exercise, and daily tasks, although some participants also noted its inadequacy relative to in-person socialization. It is possible that higher pre-pandemic computer time as measured here may be reflective of more news consumption (a stressor repeatedly noted qualitatively in our sample) or other unmeasured factors. Future exploration of this finding is needed.

Being married or partnered, female, a never-smoker, and having lower pre-pandemic sleep disturbance were associated with a more social support mid-pandemic. It is well documented in the aging literature that women report higher social support on average than men outside the pandemic context [66]. Similarly, living with a spouse or partner is a primary source of social support among many older adults, with married/partnered older adults typically reporting higher social support than those who are unmarried, widowed, or divorced [67]. Some evidence has suggested that being married or partnered may confer specific types of social support that help mitigate behaviors such as rumination, and this support may be associated with better sleep quality [68]. More broadly, growing evidence has linked better sleep quality with social support across settings, though this relationship and potential underlying mechanisms are not well understood [69]. Our findings that both being partnered and having better pre-pandemic sleep quality were associated with more perceived pandemic social support may be interrelated and warrant further exploration. Qualitative findings generally supported these quantitative findings. However, for a small subset of participants dealing with stressors in their home or marriage, such as a spouse with a severe illness, less access to external social supports due to the pandemic may have added stress rather than being supportive. Similar phenomena have been documented elsewhere in the pandemic literature [70, 71], and future studies of the relationship between marital status and social support during periods of isolation should consider collecting discrete measures of such stressors.

Better health, higher physical function, working for pay (versus being retired), more walking, less computer time, and lower sleep disturbance pre-pandemic were all associated with less fatigue mid-pandemic, suggesting promotion of resilience. For older adults outside the pandemic context, poorer health, less physical activity and more interrupted or disturbed sleep are all well documented contributors to daytime fatigue in older adults [72]. Less evidence is available for the relationship between retirement status and fatigue outside the pandemic, though one study suggested that retirement may promote decreased fatigue, particularly for older adults with chronic conditions [73]. This is counter to our finding that working prior to the pandemic was associated with less pandemic fatigue. Based on findings in the qualitative data, this difference could be driven by the pandemic-related acute loss of daily structure and social interaction, factors that have been linked to resiliency in physical health and quality of life outside of the pandemic setting [74, 75], which may have been exaggerated for participants who no longer worked. Further investigation into the relationship between retirement status and fatigue in the context of community-level stressors, like the COVID-19 pandemic, is warranted.

### Strengths & limitations

This study has several strengths. It leverages a large sample with longitudinal assessment of mental health, wellbeing, and lifestyle behaviors, allowing within person comparison of pre-pandemic and mid-pandemic levels. Most studies of older adult pandemic impacts have been cross-sectional [7, 25]. The mid-pandemic measurements used in this study were collected more than 1 year into the pandemic period, much further into the pandemic than most current literature. As such the presented findings may be more indicative of longer-term impacts of the COVID-19 pandemic. The collection and analysis of free text data from the ACT COVID survey provided unique context and insights to the presented quantitative findings.

This study has several important limitations. Generalizability of findings is limited by the overall demographic makeup of the ACT Study, which is largely Non-Hispanic White, highly educated, and residing in the Seattle, WA region. Generalizability is further limited due to survey non-response. Non-responders on average were older, less educated, and had more health and physical limitations. Furthermore, pandemic responses and societal impacts varied greatly by geographic locations within the US and between countries, and our sample is drawn from a single urban area of the US. Findings should be interpreted with caution and may represent the experience of highly educated, healthy, and Non-Hispanic White older adults from the Seattle, WA area. Another important limitation is the use of self-reported sleep, physical activity, and sedentary behavior measures, which are known to be less accurate than objective, device-based measures [76, 77]. Furthermore, while data collection for pre-pandemic lifestyle behaviors was via self-administered paper survey, data collection mode for mental health outcomes varied between pre-pandemic (face-to-face interview) and mid-pandemic (self-administered online or staff-administered phone survey) measurement. This introduces the possibility of bias due to differential mode of administration [78]; however, similar measures of health-related quality of life have been shown to be relatively robust to mode bias [79]. Given known discrepancies between objective and self-report activity data, future analyses that use objective measures of these behaviors, particularly in combination with more detailed self-assessments of activities (e.g., TV time, computer time, etc.) are needed to fully understand the relationship between these behaviors and resilience during times of societal stress and social isolation [80–82].

## Conclusions

Overall, these findings suggest several demographic, health, and lifestyle behaviors that may be protective against depressive symptoms, loss of social support, and fatigue during stressor events, like the COVID-19 pandemic. Due to the impacts of climate change and associated changes in land use and subsequent zoonotic disease spillover events, epidemic models predict the world may experience pandemic events at a higher frequency than we have historically [83, 84]. Demographic and health-related predictors explored here, such as gender, marital and retirement status, and health and function-related indicators may help researchers and public health professionals identify older adult subgroups most susceptible to harm from such events, allowing for more targeted outreach and support. For instance, being female and married/partnered were associated with resilience in pandemic social support, suggesting males and unpartnered individuals may particularly benefit from more social support resources in general, but particularly during future pandemic or other community-level stressor events. Similarly, modifiable lifestyle behaviors, such as sleep, computer usage, and physical activity could be leveraged as part of active coping strategies, previously associated with resilience to stress [85], and may represent important targets for intervention both before and during future community-level stressor events. For instance, interventions promoting improved sleep quality may support resilience across all three outcomes explored here during a future stressor event. Interventions targeting increased physical activity and decreased sedentary activities, like seated computer usage, may also be important targets for promoting resilience to fatigue and depressive symptoms. Future research further exploring these relationships and testing potential interventions targeting these modifiable behaviors is warranted.

### Supplementary Information

Supplementary Material 1. Supplemental Tables. Includes four supplemental tables supporting the analysis: 1) a table displaying search terms used in the qualitative analysis, 2) a table displaying demographic characteristics for the complete case sample used in each model, 3) modeling results from Model 1, adjusted only for demographic and health characteristics, and 4) results from a sensitivity analysis repeating fully adjusted models, additionally adjusting for baseline pre-pandemic levels of the outcome measures of interest.

## Data Availability

Data from this analysis cannot be made publicly available for ethical and legal reasons. In order to replicate our findings, a researcher may need access to personal health identifiers (PHI) including dates of birth and death and ages over 89 years. These are required variables for the analysis, and we cannot publicly release this information without IRB approval and a Data Use Agreement with interested researchers. However, the datasets used and/or analyzed in the current study are available upon reasonable request and execution of appropriate human subjects review and data sharing agreements by following the process described on the Adult Changes in Thought (ACT) website: actagingresearch.org.

## Abbreviations

ACT: Adult Changes in Thought Study
ADLs: Activities of daily living
BMI: Body mass index
CCI: Charlson Comorbidity Index
CES-D: Center for Epidemiologic Studies Depression
CHAMPS: Community Healthy Activities Model Program for Seniors
h: hours
IADLs: Instrumental activities of daily living
ISEL: Interpersonal Support Evaluation List,
KPWA: Kaiser Permanente Washignton
PROMIS: Patient-Reported Outcomes Measurement Information System
SD: Standard deviation
SF-36: Rand 36-item Short Form
