# Supplementary material for: Understanding resilience: Lifestyle-based behavioral predictors of mental health and well-being in community-dwelling older adults during the COVID-19 pandemic
Source: BMC Geriatr. 2024 Aug 12;24:676. doi: 10.1186/s12877-024-05251-3 (PMC11318348; doi:10.1186/s12877-024-05251-3)
Supplement: Supplementary file 1 — Supplementary Material 1. Supplemental Tables. Includes four supplemental tables supporting the analysis: 1) a table displaying search terms used in the qualitative analysis, 2) a table displaying demographic characteristics for the complete case sample used in each model, 3) modeling results from Model 1, adjusted only for demographic and health characteristics, and 4) results from a sensitivity analysis repeating fully adjusted models, additionally adjusting for baseline pre-pandemic levels of the outcome measures of interest. [file 12877_2024_5251_MOESM1_ESM.docx]

**Additional File 1 – Supplemental Tables**

**Supplemental Table 1.** Search terms used for preliminary coding

| **Topic** | **Keywords used to tag** | **Sub-topic and keywords** |
| --- | --- | --- |
| **Technology and Computer Time** | Technology; virtual; computer; online; internet; zoom; phone; video; chat; text; message; social media | **Purpose**: social, physical, independent entertainment, other |
| **Television Time** | TV; movie; show; television; streaming | **Sub-activity:** More TV/at home movies; no more going to movies; TV for exercise |
| **Sleep time and quality** | sleep; fall asleep; nap; drowse; insomnia; rest; restlessness |  |
| **Total sitting** | sit; sedentary; mobile; chair; couch; sofa; move |  |
| **Physical activity/exercise** | activity; exercise; workout; walk; zumba; yoga; swim; dance; silver sneakers; fitness; jazzercise; move; hike; gym; pool; bike; cycle; PT; OT; garden; yard | **Amount**: more, less, no change, no activity |
| **Depressive symptoms** | depression; sad; down; low; mental health; helpless; sorrow; misery; unhappy |  |
| **Loneliness** | loneliness; isolated; alone; secluded; solitary; separate |  |
| **Social Support** | social support; friend; family; neighbor; community; coworker; church; temple; synagogue; club; <various relational names> | **Valence**: positive (improved, more, new) vs. negative (less, none, loss) |
| **Fatigue** | fatigue; tired; energy |  |
| **Anxiety/Stress** | anxiety; stress; worry |  |
| **Loss** | miss - sort into above categories |  |

**Supplemental Table 2.** Baseline demographics & pre-pandemic characteristics of complete case groups for each outcome analysis

|  | **Depressive Symptoms** | | **Social Support** | | **Fatigue** | |
| --- | --- | --- | --- | --- | --- | --- |
|  | Model 1 | Model 2 | Model 1 | Model 2 | Model 1 | Model 2 |
|  | N=792 | N=691 | N=774 | N=675 | N=806 | N=703 |
|  | **mean (SD)** | **mean (SD)** | **mean (SD)** | **mean (SD)** | **mean (SD)** | **mean (SD)** |
|  | **%** | **%** | **%** | **%** | **%** | **%** |
| **Age (pre-COVID), years** | 76.9 (6.5) | 76.6 (6.3) | 76.8 (6.4) | 76.4 (6.2) | 77.0 (6.5) | 76.7 (6.4) |
| **Time between measures, years** | 3.0 (1.3) | 3.0 (1.3) | 3.0 (1.3) | 3.0 (1.3) | 3.0 (1.3) | 3.0 (1.3) |
| **Sex** |  |  |  |  |  |  |
| Male | 42.6 | 43.9 | 43.0 | 44.3 | 42.4 | 43.7 |
| Female | 57.5 | 56.2 | 57 | 55.7 | 57.6 | 56.3 |
| **Race** |  |  |  |  |  |  |
| Asian | 2.8 | 3.0 | 2.7 | 3.0 | 3.1 | 3.3 |
| Black | 1.6 | 1.6 | 1.8 | 1.8 | 1.7 | 1.7 |
| Other | 5.6 | 5.9 | 5.7 | 6.1 | 5.5 | 5.8 |
| White | 90.0 | 89.4 | 89.8 | 89.2 | 89.7 | 89.2 |
| **Ethnicity** |  |  |  |  |  |  |
| Non-Hispanic/Latinx | 98.4 | 98.4 | 98.5 | 98.5 | 98.4 | 98.4 |
| Hispanic/Latinx | 1.6 | 1.6 | 1.6 | 1.5 | 1.6 | 1.6 |
| **Education (years)** | 17.2 (2.6) | 17.2 (2.5) | 17.2 (2.6) | 17.3 (2.5) | 17.2 (2.6) | 17.2 (2.5) |
| **Currently work for pay** | 16.9 | 17.1 | 17.3 | 17.3 | 17.0 | 17.1 |
| **Marital Status** |  |  |  |  |  |  |
| Single/Divorced/Widowed | 39.5 | 37.3 | 39.0 | 37.0 | 40.0 | 38.0 |
| Married/Partnered | 60.5 | 62.7 | 61.0 | 63.0 | 60.1 | 62.0 |
| **Living Arrangement** |  |  |  |  |  |  |
| Live Alone | 33.0 | 32.6 | 32.7 | 32.4 | 33.4 | 33.1 |
| Live w/ Partner, Relative, Friend | 66.5 | 66.9 | 66.8 | 67.0 | 66.1 | 66.3 |
| Live in Adult Family Home or Nursing Home | 0.5 | 0.6 | 0.5 | 0.6 | 0.5 | 0.6 |
| **BMI** | 27.1 (5.1) | 27.2 (5.2) | 27.1 (5.1) | 27.2 (5.2) | 27.1 (5.1) | 27.2 (5.2) |
| **Charlson Comorbidity Index^1^** | 0.95 (1.51) | 0.90 (1.46) | 0.95 (1.50) | 0.90 (1.45) | 0.97 (1.47) | 0.92 (1.47) |
| **Smoking Status** |  |  |  |  |  |  |
| Never | 92.6 | 92.3 | 92.4 | 92.2 | 92.6 | 92.3 |
| Former | 5.9 | 6.1 | 5.9 | 6.1 | 5.8 | 6.0 |
| Current | 1.5 | 1.6 | 1.7 | 1.8 | 1.6 | 1.7 |
| **Number ADLs with Difficulty^2^** |  |  |  |  |  |  |
| None | 81.3 | 82.3 | 81.9 | 83.1 | 81.4 | 82.4 |
| 1 or more | 18.7 | 17.7 | 18.1 | 16.9 | 18.6 | 17.6 |
| **Number IADLs with difficulty^2^** |  |  |  |  |  |  |
| None | 86.1 | 86.3 | 86.3 | 86.5 | 86.1 | 86.2 |
| 1 or more | 13.9 | 13.8 | 13.7 | 13.5 | 13.9 | 13.8 |
| **Pre-COVID Daily Total Sitting^3^** | 7.87 (2.93) | 7.86 (2.91) | 7.89 (2.93) | 7.88 (2.91) | 7.89 (2.92) | 7.88 (2.90) |
| **Pre-COVID Daily TV time^3^** | 2.57 (1.71) | 2.53 (1.68) | 2.61 (1.73) | 2.58 (1.71) | 2.59 (1.72) | 2.56 (1.69) |
| **Pre-COVID Daily Computer Time^3^** | 1.86 (1.62) | 1.87 (1.60) | 1.86 (1.62) | 1.88 (1.59) | 1.86 (1.62) | 1.88 (1.59) |
| **Pre-COVID Days/week Walking For Exercise** | 1.61 (1.88) | 1.62 (1.88) | 1.61 (1.87) | 1.62 (1.87) | 1.59 (1.88) | 1.60 (1.87) |
| **Pre-COVID Sleep Disturbance (PROMIS T-score)** | 46.80 (7.81) | 46.94 (7.80) | 46.70 (7.78) | 46.85 (7.76) | 46.76 (7.88) | 46.90 (7.88) |
| **Post-COVID Social Support Scale (ISEL) Score**^4^ | 21.42 (2.86) | 21.44 (2.88) | 21.43 (2.86) | 21.45 (2.88) | 21.43 (2.86) | 21.45 (2.88) |
| **Post-COVID Depressive Symptoms (CES-D 10-item Score)** | 5.14 (4.79) | 5.07 (4.65) | 5.08 (4.80) | 5.02 (4.67) | 5.14 (4.79) | 5.07 (4.65) |
| **Post-COVID Energy/Fatigue (SF-36 sub-scale score)** | 57.67 (19.71) | 57.72 (19.79) | 57.73 (19.63) | 57.82 (19.69) | 57.49 (19.79) | 57.54 (19.86) |

^1^CCI is set to missing for individuals without current enrollment data or without a visit in the prior year.

^2^ADLs: Walking around the house, Getting out of bed/chair, Feeding oneself, Dressing oneself, Bathing/showering oneself, Getting to or using the toilet. IADLs: Light housework, Shopping for personal items, Preparing meals, Managing money/paying bills, Using telephone.

**^3^**Self-reported time was reported in categorical variables by approximate number of total hours (e.g. 1-2 h, 2-3 h, etc.) and transformed to an approximate total number of hours by assigning the midpoint of the chosen range. More details available in the Methods section.

^4^Modified ISEL score calculated as the sum of the 6 collected items, shortened from the original scale's 12. Range =6-24, higher score denote more social support.

Abbreviations: SD = standard deviation, BMI = body mass index, ADLs = activities of daily living, IADLs = instrumental activities of daily living; PROMIS = Patient-Reported Outcomes Measurement Information System, ISEL = Interpersonal Support Evaluation List, CES-D = Center for Epidemiologic Studies Depression, SF-36 = Rand 36-item Short Form.

**Supplemental Table 3.** Model 1 (demographic and health predictors only) results for all mental health and wellbeing outcomes of interest

|  | **A. Pandemic Depressive Symptoms** | | | **B. Pandemic Social Support** | | | **C. Pandemic Fatigue^2^** | | |
| --- | --- | --- | --- | --- | --- | --- | --- | --- | --- |
|  | β | 95% CI | | β | 95% CI | | β | 95% CI | |
|  | N=792 |  |  | N=774 |  |  | N=806 |  |  |
| **Age (y)^1^** | -0.03 | -0.09 | 0.03 | **0.05** | **0.01** | **0.08** | -0.16 | -0.38 | 0.07 |
| **Male Sex** | **-0.90** | **-1.60** | **-0.21** | **-0.44** | **-0.86** | **-0.01** | 2.53 | -0.24 | 5.31 |
| **Non-White race** | 0.15 | -0.95 | 1.26 | -0.41 | -1.08 | 0.26 | -0.38 | -4.73 | 3.97 |
| **Hispanic Ethnicity** | 0.19 | -2.40 | 2.78 | 0.62 | -1.01 | 2.25 | -2.77 | -13.17 | 7.63 |
| **Years Education^1^** | 0.12 | -0.01 | 0.25 | 0.01 | -0.07 | 0.09 | -0.26 | -0.77 | 0.25 |
| **Currently Work for Pay** | -0.72 | -1.62 | 0.19 | -0.12 | -0.67 | 0.43 | 2.88 | -0.72 | 6.47 |
| **Married/Partnered** | **-1.24** | **-2.42** | **-0.06** | 0.62 | -0.11 | 1.35 | -0.24 | -4.95 | 4.47 |
| **Live Alone** | -0.09 | -1.28 | 1.10 | -0.18 | -0.92 | 0.55 | -1.42 | -6.16 | 3.31 |
| **BMI^1^** | 0.05 | -0.02 | 0.11 | -0.01 | -0.05 | 0.04 | **-0.52** | **-0.79** | **-0.25** |
| **CCI^1^** | 0.19 | -0.03 | 0.42 | -0.05 | -0.19 | 0.09 | **-1.38** | **-2.28** | **-0.47** |
| **Ever Smoker** | **2.09** | **0.80** | **3.39** | **-0.87** | **-1.65** | **-0.08** | **-6.16** | **-11.32** | **-0.99** |
| **ADLs with difficulty** | **0.78** | **0.16** | **1.41** | **-0.47** | **-0.86** | **-0.09** | **-4.02** | **-6.48** | **-1.55** |
| **IADLs with difficulty** | 0.36 | -0.27 | 1.00 | 0.07 | -0.32 | 0.46 | **-4.38** | **-6.92** | **-1.85** |
| **Survey month** | 0.17 | -0.20 | 0.54 | 0.14 | -0.08 | 0.37 | -0.48 | -1.96 | 1.00 |
| **Constant** | **5.21** | **4.96** | **7.27** | **20.90** | **19.64** | **22.17** | **61.11** | **52.91** | **69.32** |

^1^Variables were centered at the sample mean value for modeling to allow interpretation of the intercept values at those sample means. Centering values are as follows: age=77.17746, years education=17.08817, BMI=27.06915, CCI= 0.9731308.

^2^Higher scores on the SF-36 denote more favorable health states. Here, higher scores indicate less fatigue (more energy).

Abbreviations: BMI = body mass index, CCI = Charlson Comorbidity Index, ADLs = activities of daily living, IADLs = instrumental activities of daily living

**Supplemental Table 4.** Sensitivity analysis modeling results adjusting for baseline levels of mental health and wellbeing outcomes of interest

|  | **Pandemic Depressive Symptoms** | | | **Pandemic Social Support** | | | **Pandemic Fatigue^3^** | | |
| --- | --- | --- | --- | --- | --- | --- | --- | --- | --- |
|  | β | 95% CI | | β | 95% CI | | β | 95% CI | |
|  | N=688 |  |  | N=671 |  |  | N=697 |  |  |
| **Age (y)^1^** | 0.01 | -0.05 | 0.06 | 0.03 | -0.00 | 0.06 | -0.02 | -0.22 | 0.18 |
| **Male Sex** | -0.37 | -0.97 | 0.23 | -0.13 | -0.48 | 0.22 | 2.03 | -0.28 | 4.34 |
| **Non-White race** | -0.86 | -1.80 | 0.08 | -0.07 | -0.63 | 0.48 | -0.49 | -4.10 | 3.11 |
| **Hispanic Ethnicity** | 2.16 | -0.12 | 4.43 | -0.08 | -1.56 | 1.39 | -2.51 | -11.33 | 6.31 |
| **Years Education^1^** | 0.06 | -0.07 | 0.18 | -0.03 | -0.10 | 0.39 | -0.42 | -0.89 | 0.04 |
| **Currently Work for Pay** | -0.56 | -1.36 | 0.23 | -0.13 | -0.60 | 0.34 | 2.58 | -0.50 | 5.65 |
| **Married/Partnered** | -0.85 | -1.94 | 0.23 | 0.55 | -0.10 | 1.21 | -2.93 | -7.10 | 1.24 |
| **Live Alone** | -0.77 | -1.86 | 0.33 | 0.27 | -0.40 | 1.21 | -4.08 | -8.29 | 0.13 |
| **BMI^1^** | 0.02 | -0.04 | 0.08 | -0.01 | -0.04 | 0.03 | -0.15 | -0.38 | 0.08 |
| **CCI^1^** | 0.09 | -0.11 | 0.30 | -0.01 | -0.13 | 0.11 | 0.09 | -0.70 | 0.88 |
| **Ever Smoker** | **1.66** | **0.56** | **2.77** | -0.32 | -0.97 | 0.33 | **-6.30** | **-10.54** | **-2.06** |
| **ADLs with difficulty** | 0.16 | -0.40 | 0.72 | -0.13 | -0.46 | 0.20 | -0.67 | -2.81 | 1.46 |
| **IADLs with difficulty** | -0.03 | -0.61 | 0.55 | 0.11 | -0.24 | 0.45 | **-2.31** | **-4.54** | **-0.09** |
| **Pre-Pandemic Total Daily sitting (h)^1,2^** | -0.03 | -0.13 | 0.08 | -0.02 | -0.08 | 0.04 | 0.30 | -0.11 | 0.70 |
| **Pre-Pandemic TV time (h)^1,2^** | -0.02 | -0.20 | 0.17 | -0.05 | -0.16 | 0.05 | -0.26 | -0.96 | 0.45 |
| **Pre-Pandemic Computer Time (h)^1,2^** | 0.14 | -0.06 | 0.34 | -0.03 | -0.15 | 0.08 | **-0.85** | **-1.61** | **-0.09** |
| **Pre-Pandemic Walking (days)^1^** | -0.11 | -0.27 | 0.05 | -0.02 | -0.11 | 0.08 | 0.31 | -0.30 | 0.92 |
| **Pre-Pandemic Sleep Disturbance (PROMIS score)^,^** | **0.09** | **0.05** | **0.13** | 0.00 | -0.02 | 0.02 | -0.03 | -0.18 | 0.12 |
| **Pre-Pandemic Social Support^1^** | - | - | - | **0.97** | **0.88** | **1.07** | - | - | - |
| **Pre-Pandemic Depressive Symptoms (CES-D)^1^** | **0.60** | **0.52** | **0.68** | - | - | - | - | - | - |
| **Pre-Pandemic Fatigue (SF-36)^1^** | - | - | - | - | - | - | **0.74** | **0.67** | **0.81** |
| **Survey month** | 0.04 | -0.28 | 0.36 | 0.00 | -0.19 | 0.19 | -0.75 | -1.98 | 0.49 |
| **Constant** | **5.90** | **4.07** | **7.72** | **21.10** | **20.01** | **22.19** | **63.63** | **56.61** | **70.65** |

^1^Variables were centered at the sample mean value for modeling to allow interpretation of the intercept values at those sample means. Centering values are as follows: age=77.17746, years education=17.08817, BMI=27.06915, CCI= 0.9731308 , pre-pandemic total sitting=7.893868, pre-pandemic TV time=2.592466, pre-pandemic computer time=1.851947, pre-pandemic walking=1.558962, pre-pandemic sleep disturbance=46.78341, pre-pandemic social support= 22.68462, pre-pandemic depressive symptoms=3.69843, and pre-pandemic fatigue=62.27409.

^2^Self-reported time was reported in categorical variables by approximate number of total hrs (e.g. 1-2 hrs, 2-3 hrs, etc.) and transformed to an approximate total number of hours as described in the methods section.

^3^Higher scores on the SF-36 denote more favorable health states. Here, higher scores indicate less fatigue (more energy).

Abbreviations: BMI = body mass index, CCI – Charlson Comorbidity Index, ADLs = activities of daily living, IADLs = instrumental activities of daily living

**Supplemental Table 5.** Correlation matrix of independent variables included in regression modeling

|  | **Age** | **Sex** | **Race** | **Ethnicity** | **Education** | **Currently Work for Pay** | **Married/ Partnered** | **Live Alone** | **BMI** | **CCI** | **Ever Smoke** | **ADLs** | **IADLs** | **Survey month** | **Daily Sitting** | **TV time** | **Computer Time** | **Walking** | **Sleep** |
| --- | --- | --- | --- | --- | --- | --- | --- | --- | --- | --- | --- | --- | --- | --- | --- | --- | --- | --- | --- |
| **Age** | 1 |  |  |  |  |  |  |  |  |  |  |  |  |  |  |  |  |  |  |
| **Sex** | -0.059 | 1 |  |  |  |  |  |  |  |  |  |  |  |  |  |  |  |  |  |
| **Race** | 0.0268 | -0.046 | 1 |  |  |  |  |  |  |  |  |  |  |  |  |  |  |  |  |
| **Ethnicity** | -0.0076 | -0.024 | 0.1255 | 1 |  |  |  |  |  |  |  |  |  |  |  |  |  |  |  |
| **Education** | -0.1505 | 0.1995 | 0.0072 | -0.0114 | 1 |  |  |  |  |  |  |  |  |  |  |  |  |  |  |
| **Currently Work for Pay** | -0.241 | 0.0819 | -0.0418 | 0.0557 | 0.074 | 1 |  |  |  |  |  |  |  |  |  |  |  |  |  |
| **Married/ Partnered** | -0.259 | 0.257 | -0.1058 | 0.0318 | 0.1377 | 0.083 | 1 |  |  |  |  |  |  |  |  |  |  |  |  |
| **Live Alone** | 0.1926 | -0.2 | 0.0702 | -0.04 | -0.1152 | -0.0691 | -0.8137 | 1 |  |  |  |  |  |  |  |  |  |  |  |
| **BMI** | -0.0942 | 0.1096 | -0.0542 | -0.0123 | -0.0525 | 0.0505 | -0.0187 | 0.0208 | 1 |  |  |  |  |  |  |  |  |  |  |
| **CCI** | 0.2212 | 0.0137 | 0.0702 | -0.0334 | -0.0984 | -0.0411 | -0.1342 | 0.072 | 0.1406 | 1 |  |  |  |  |  |  |  |  |  |
| **Ever Smoke** | -0.2534 | 0.0212 | 0.0573 | -0.0025 | -0.0417 | 0.1463 | 0.0097 | -0.022 | 0.0935 | -0.034 | 1 |  |  |  |  |  |  |  |  |
| **ADLs with difficulty** | 0.1178 | 0.0135 | 0.0395 | -0.001 | -0.0295 | -0.0124 | -0.0874 | 0.0682 | 0.2358 | 0.1771 | -0.0041 | 1 |  |  |  |  |  |  |  |
| **IADLs with difficulty** | 0.1439 | 0.0021 | 0.0147 | -0.0013 | -0.0351 | -0.0613 | -0.0654 | 0.0637 | 0.1123 | 0.1102 | -0.0095 | 0.5203 | 1 |  |  |  |  |  |  |
| **Survey month** | -0.0811 | -0.046 | -0.0161 | -0.0513 | -0.0441 | -0.0049 | -0.0293 | -0.022 | -0.05 | -0.003 | 0.0315 | -0.0313 | -0.0143 | 1 |  |  |  |  |  |
| **Daily sitting** | 0.0693 | 0.0699 | -0.0117 | -0.0113 | 0.0736 | 0.0186 | -0.0542 | 0.011 | 0.1652 | 0.1055 | -0.0162 | 0.1622 | 0.1579 | -0.0442 | 1 |  |  |  |  |
| **TV time** | 0.1707 | -0.04 | 0.1068 | -0.0117 | -0.241 | -0.0654 | -0.097 | 0.0946 | 0.1853 | 0.1967 | -0.0175 | 0.0998 | 0.0529 | -0.0119 | 0.2206 | 1 |  |  |  |
| **Computer Time** | -0.2198 | 0.0938 | 0.0686 | -0.0123 | 0.1885 | 0.232 | 0.0116 | 0.0115 | 0.0547 | -0.029 | 0.1078 | 0.0138 | -0.0472 | -0.0501 | 0.2395 | -0.0369 | 1 |  |  |
| **Walking** | -0.1305 | -0.038 | -0.0799 | 0.008 | 0.0326 | 0.0023 | 0.0881 | -0.043 | -0.1759 | -0.14 | 0.0355 | -0.1831 | -0.1244 | 0.0271 | -0.1599 | -0.1137 | -0.0258 | 1 |  |
| **Sleep** | 0.023 | -0.115 | 0.0745 | 0.0046 | -0.0675 | -0.0943 | -0.1237 | 0.0941 | 0.1264 | 0.0437 | 0.0101 | 0.1815 | 0.1478 | 0.0256 | 0.0954 | 0.0888 | 0.051 | -0.0516 | 1 |
